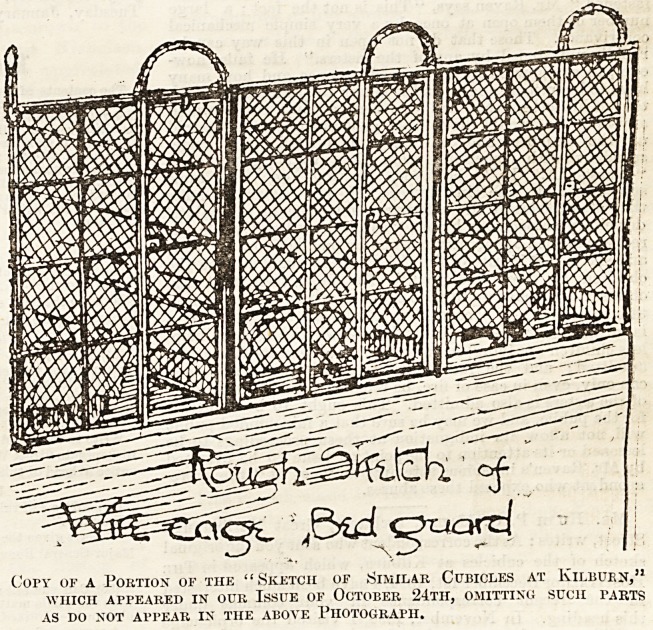# The Hospital Nursing Supplement

**Published:** 1896-12-05

**Authors:** 


"The Hospital" Nursing Mirror.

Being the Extra Nursing Supplement of "The Hospital."

[Contributions for this Supplement should be addressed to the Editor, The Hospital, 28 & 29, Southampton Street, Strand, London, W.C., and should have the word "Nursing" plainly written in left-hand top corner of the envelope.]

### News from the Nursing World.

### PRINCESS LOUISE IN SCOTLAND.

Princess Louise, Marchioness of Lorne, opened an addition to the Home of the Queen's Jubilee Nurses' Institute, at Castle Terrace, Edinburgh, on November 23rd. Her Royal Highness was accompanied by the Marquis of Lorne, who, after the new premises had been declared open, made a brief speech. A good many people interested in the work of Queen's Nurses in Scotland were present, including the Lord Provost, Lord Reay, Sir Thomas Clark, and Mr. Cox, M.P. Lord Reay stated that fourteen branches had been affiliated this year, making the total number of associations now joined to the institute seventy-two.

### NEEDLEWORK EXHIBITION AT FROGMORE.

Princess Henry of Battenberg is President of the Berks and Bucks Needlework Guild, and last week Her Royal Highness presided over an exhibition at Frogmore House of the work done during the year by its members. Poor parishes, hospitals, and other charities in the two counties will benefit by the distribution of the warm and useful clothing thus provided.

### NURSING UNDER THE METROPOLITAN ASYLUMS BOARD.

At a recent meeting of the Metropolitan Asylums Board the following amendment to a recommendation submitted to the Board by the General Purposes Committee in October, regarding nursing in the asylums under the management of the Board, was discussed and carried: "That the recommendation proposing 'that a trained hospital nurse, with the title of Superintendent Nurse, be placed in responsible charge of the nursing of all female infirmary patients at each of the managers' asylums' be not adopted, but that it be an instruction to the committee of management of the Imbecile Asylums to appoint a trained hospital nurse, with the title of Assistant Matron, who shall control and superintend the nursing of both male and female infirmaries, and do the skilled nursing herself when required."

### WOMEN GUARDIANS ON WORKHOUSE NURSING.

Mrs. Johnstone and Mrs. J. H. Radford, of the Tavistock Board of Guardians, have been making earnest attempts to secure a properly trained second nurse for the Tavistock Union Infirmary, through the Workhouse Infirmary Nursing Association. In spite of the unanimous recommendation of the Nursing Committee that a certificated nurse should be appointed, the fact that a resolution to that effect was actually passed at a meeting of the board last September, and the strong and sensible remarks made by the two ladies, as well as by other guardians, at a recent meeting, on the motion of the Rev. Dr. Bryant, an amendment was passed by seventeen votes to fifteen, rescinding the former resolution and resolving that an assistant nurse should be advertised for, "the mover remarking that he would not say whether she should be trained or not. The present nurse, who is untrained, has now between fifty and sixty patients under her care. Such a retrograde step on the part of the Tavistock Board is deplorable, and the fifteen enlightened guardians who voted on the side of humanity and progress have our sincere sympathy in their defeat.

### CHRISTMAS GIFTS FOR THE HOSPITALS.

We have to acknowledge, with many thanks, the receipt of a welcome order for 5s. from Miss Alice M. Newson, a former contributor to our Christmas Clothing Fund, to be expended in the purchase of warm garments. We hope our readers are doing their very best to collect nice things for the distribution this year, and urging their friends by example and precept to send in contributions in kind or money. All contributions should be sent to The Hospital Office, 28 and 29, Southampton Street, Strand, London, W.C., not later than December 16th.

### THE BENSON DISTRICT NURSING HOME.

An appeal is being made by the honorary secretary of this home, which was started partly as a memorial to the daughter of the late Archbishop of Canterbury, and is partly maintained by voluntary subscription, for money to clear the institution from debt and set it upon a firm basis. Its object is to supply skilled nursing to the poor in the forlorn districts of Southwark, Newington, and Walworth, irrespective of religious denomination. The area worked by the present staff of four nurses and superintendent consists of sixteen parishes, one of which alone contains 12,000 inhabitants. All who know the poverty and wretchedness which abound in these districts of Southern London will realise the regret which will be felt should it be found necessary to curtail the work for want of funds. Annual subscriptions are much needed, and any help will be gladly received by the treasurer, Miss Lucy Fowler, 44, Nelson Square, Blackfriars Road, S.E.

### A "SCHOLAR'S COT" FOR BRADFORD INFIRMARY.

The board of management of the Bradford Infirmary have decided to appeal to "The Children of Bradford" to commemorate the sixtieth year of the Queen's reign by establishing and endowing a free cot in the children's ward, to be known as "The Scholar's Cot." For this at least a sum of £1,200 must be subscribed, and a committee has been appointed "representative of the educational interests of the town" to make the scheme known to all children attending Sunday and day schools. Collecting cards will be issued, and it is hoped that the general public of Bradford will do their best to further the movement, and to enlist the children's sympathies on behalf of the little ones who in times of sickness are dependent on the help afforded by hospital treatment. About sixty beds in the infirmary are always occupied by children, and the number treated as in-patients has increased largely during the last few years.

### A GIFT TO THE ROYAL ALBERT ASYLUM, LANCASTER.

At the meeting of the Central Committee on Friday last, the Right Hon. Sir John T. Hibbert (chairman) presiding, Sir Thomas Storey, of Lancaster, offered to build on the asylum estate a home for forty feeble-minded girls who have been trained in the institution, and who will be occupied in its domestic and nursing service. Sir Thomas Storey's generous offer was gratefully accepted, and it was unanimously agreed to call the home "The Storey Home for Feeble-minded Girls, in connection with the Royal Albert Asylum."

### MEDALS FOR BATH NURSES.

The President of the Royal United Hospital, Bath, the Rev. Edward Handley, who has been a warm friend to that institution for many years, has undertaken to present gold and silver medals each year to the two nurses who have shown themselves the most competent during their three years' training. The general conduct and efficiency of the nurses as shown by the matron's reports are important qualifications. The maximum number of marks to be gained is four hundred—one hundred each for medical and surgical nursing, and two hundred for "general conduct." The first two medals have just been presented; the gold medal to Nurse Rachel Pryce Jones, and the silver one to Nurse Mary Alabaster, the total number of candidates being eight. The medals are handsome in design, carrying on one side the name of the winner and on the other a reproduction of the seal of the hospital.

### TIVERTON INFIRMARY.

Miss Reilly's departure from Tiverton Infirmary, where for eight years she has held the post of matron, has been much regretted, and before leaving she was presented with a purse containing gold and an illuminated address, expressing sorrow at the loss of one who has won the esteem and regard of all who have come into contact with her, and who have experienced her ever-ready sympathy and desire to aid. Miss Reilly received a silver tea service and other gifts from friends, nurses, and patients, and carries away with her many hearty good wishes.

### THE ROYAL NATIONAL PENSION FUND FOR NURSES.

The spontaneous effort on the part of the nurses to commemorate the sixtieth year of her Majesty's reign by increasing the funds of the Junius S. Morgan Benevolent Fund is making splendid progress. It is most gratifying to find how large a proportion of the whole number of nurses have gladly consented to subscribe themselves from 1s. per annum upwards to this Fund, and it is further worthy of praise that the people under whose notice the nurses have brought it have readily and generously added their contributions. One district nurse at Newton Abbot writes: "I cannot say I have found the task disagreeable; quite the contrary. I have thoroughly enjoyed it, and shall never forget the kind reception and hospitality I have received from house to house from comparative strangers." We have great pleasure in giving publicity to this generosity on the part of the inhabitants of Newton Abbot to their district nurse, whom they have so kindly treated, and of whom they are deservedly proud. The sum the district nurse at Newton Abbot has already raised amounts to £20 4s. 6d. We have once more to request that every Pension Fund nurse who has not yet replied to the secretary's letter will do so without a day's further delay, especially if she does not see her way either to contribute herself or to endeavour to induce others to take this course.

### WOMEN IN AUSTRIA.

Baron Gautsch, the Austrian Minister of Instruction, has announced that next year the Government intend to legalise the admission of women to all faculties of the Universities except theology. The right to practise will also be conferred upon medical women who have obtained their degrees at foreign Universities.

### NAUHEIM TREATMENT.

We have been asked by several correspondents where instruction in the Schott or Nauheim exercises may be obtained. A winter series of classes to teach the exercises and the preparation of the baths will begin at the Trained Nurses' Club, 12, Buckingham Street, as soon as the number of applications makes it worth while to arrange for them. Nurses wishing for such instruction should therefore lose no time in communicating with the hon. secretary, Society of Trained Masseuses, at the above address.

### NEW WING TO BASINGSTOKE COTTAGE HOSPITAL.

A new wing, built and furnished entirely by Lieut.-Colonel May, the Mayor of Basingstoke, has been added to the Basingstoke Cottage Hospital. The opening took place on November 16th, and was made an occasion of much rejoicing. Colonel May has been a warm friend to the hospital during the five years he has acted as mayor, which office has been filled no less than eighteen times by members of his family, the wards just opened being erected "to commemorate the election of his ancestor, Thomas May, as Mayor of Basingstoke in 1796 and his own in 1896." The new wing provides eight extra beds, in two wards, with linen stores, lavatories, bathrooms, &c. In the course of various speeches made on the opening day, many pleasant things were said of the nursing staff, who, said one speaker, "did their work well, for their hearts were in it."

### SHORT ITEMS.

The Duke of Westminster has lent a room at Grosvenor House for a preliminary meeting of the Committee of the "Queen's Commemoration Fund" on behalf of the Jubilee Institute for Nurses.—A pleasant and successful concert was given to the nurses of Guy's Hospital, in the Court Room, on the evening of November 18th, got up by the efforts of Messrs. Lidderdale and Fortescue Briskdale.—Miss Alice Oram, night superintendent of nurses at the Mile End Infirmary, E., was presented with a charming five o'clock tea service by the night staff of the union on her recently leaving the infirmary to take up a similar appointment elsewhere.—The *British Medical Journal* of November 28th says "in the case of 'Beatty v. Cullingworth,' notice has been given on the part of Miss Beatty for a new trial. The grounds of the application are not at present made public.—A special performance at the Haymarket Theatre on behalf of the Commemoration Fund for the Queen's Jubilee Institute has been promised by Messrs. Frederick Harrison and Cyril Maude during next year.—The Committee of Queen Charlotte's Lying-in Hospital have received a donation of £1,000 from the Dowager Lady Howard de Walden, through Mr. F. G. Cavendish Bentinck, towards the extension and improvement fund.—Princess Christian visited the North London Hospital for Consumption, Hampstead, on Tuesday afternoon, for the purpose of unveiling a tablet in memory of the late Mr. Rundle Charles.

### Hygiene: For Nurses.

By John Glaister, M.D., F.F.P.S.G., D.P.H.Camb., Professor of Forensic Medicine and Public Health, St. Mungo's College, Glasgow, &c.

### XXXV. — INFECTIOUS DISEASES — MODES OF SPREAD—MODES OF PREVENTION OF SPREAD —PROPHYLAXIS — NOTIFICATION — ISOLATION —QUARANTINE—DISINFECTION.

Starting from the universally acknowledged fact that infective diseases are transmissible from person to person, the liability to attack is determined, first, by the person coming within the area of infection, either from the patient or material which has been in contact with him; and second, by his predisposition, susceptibility, or vulnerability. Those infective materials which succumb quickly to fresh air and sunlight, have a limited infectivity; while, on the other hand, those which are most resistant are capable of existing in suitable media outside of the body, as in soil, food, water, &c., and have, therefore, a larger field of infectivity.

The modes by which infection may be transported are as follows: (1) By direct contact with the infective person—hence called contagion; (2) by contact with anything which has been in contact with the infective person, or which proceeds from the apartment in which he is treated; (3) by intercommunication between infected animals and man; (4) transportation by insects, as mosquitos, gnats, &c.; (5) in water or food; and (6) by the air.

Visitation to the houses of the infected sick, or the wilful exposure of children to others who are suffering from a mild type of disease (in the erroneous assumption that all children must sooner or later contract the disease, and the sooner it is over, and of a mild type, the better), is chiefly to blame for the spread of infective diseases. Advancing education, however, is altering both practices. There is no guarantee that exposure to a mild type of disease will be followed by an equally mild seizure in the exposed child, for it is a common experience that children of the same family, even, do not contract attacks of equal severity; and this is equally true of children of different parents. Under the second head, the clothing, or books, or toys of the infective person may act as vehicles of infection. While the clothing may be disinfected, it is always safer to consign to the flames such toys and books as are admitted into the sick-room. For the above reason, Public Health Acts make it penal to expose in a public place any child who is suffering from infective disease, or for any person so to expose himself, or to sell or pawn any article of infective clothing. The room occupied by the infective sick is deemed infective until disinfection has been effected, and what applies to the room applies to vehicles used for the transportation of such persons, and to the contents of the room or vehicle. Under the third head are included such diseases, already spoken of, which are communicable from animal to man. The agency of insects in the transportation of this class of diseases is now well established, particularly insects which seek their food in excrementitious matter, or which suck the blood of the person. Under the fifth head are included such infective diseases as are generated from the partaking of infected water or food. Cholera and enteric fever are typical of this class. It ought to be well known that milk, being an organic fluid, is one of the best natural absorbents of infective or septic matter, and, moreover, offers an excellent nutrient medium to many microbes. Unused milk from a sick room, therefore, ought to be treated as slops, and should not be taken by healthy persons. In impending or prevailing epidemics of cholera or enteric fever, a simple and effective plan to render milk or water harmless is to boil it before use, and to re-aerate the latter as previously described. Considering also the possibility of the transmission of scarlet fever and diphtheria by milk, this precaution becomes of greater importance. Indeed, it is a question whether it should not be done as a routine practice. The chief objection to boiled milk is its taste; this, however, would be minimised by the use of milk sterilisers, in which the milk is rendered sterile at a temperature short of boiling point. Sterilisation of milk is now carried out on the large scale in Copenhagen, Berlin, and other places on the Continent, before it is sold. The operation is easily carried out by placing the milk in a large bottle, placing the bottle in another vessel containing water, and heating until the temperature of the milk reaches 170 deg. Fahr., at which temperature it ought to be maintained for, at least, fifteen minutes; then stoppering the milk bottle, allow its contents to gradually cool, when it becomes absolutely safe for use. For children, and, indeed, for all persons who use milk largely, this practice has much to commend it. The last mode of conveying infection is by atmospheric currents. A necessary condition to infective material being air-borne is that it must exist in the dried condition. As we have pointed out, when it is enveloped in a moist or wet medium it cannot become air-borne. The convection of infective material by the air is clearly demonstrated in malaria.

The proximate modes by which infective material enters the body of a susceptible person are (1) inoculation, (2) by inhalation, (3) by absorption, and (4) by ingestion.

The means by which the spread of infective diseases is prevented are the following, viz.: (1) prophylaxis (Gr., *prophylasso*—to prevent); (2) notification; (3) isolation and quarantine; and (4) disinfection.

Prophylaxis, or the prevention of disease, is operative generally during health, but especially during the interval between a threatened epidemic and its actual outbreak. Prophylactic measures include (1) general instructions to the people as to means whereby the attack may be avoided; (2) special attention to the working efficiency of sanitary measures; and (3), if deemed advisable, the establishment of a cordon between infected and non-infected countries or districts. The other modes come into operation immediately the first case of the outbreak appears, and they aid in checking or limiting further extension of the disease.

Notification of infectious diseases supplies a powerful instrument to sanitary authorities, first, in enabling them to "locate" the area of the disease, and to ascertain the number of persons affected; and, second, to concert suitable measures of isolation and disinfection to prevent its extension. In some populous places the duty is laid solely upon the parent or guardian, or solely upon the medical attendant, while in the Notification Act of 1889 the duty is laid equally upon both, failure to notify, after knowledge of the fact, carrying with it a penalty.

Isolation and quarantine are means of prevention, which, although they do not mean the same thing, subserve the same purpose. The former means the separation of the infective sick from the healthy, in such circumstances as will prevent, as far as can be, the spread of the disease, and for such periods of time as infectivity prevails; quarantine, the separation from the healthy of persons or things which, although themselves not yet actively infected, may be the passive carriers of infection from an infected area, for such space of time as will demonstrate whether or not the former will become actively infected, and as will enable disinfection to be employed in respect of the latter. The practice of quarantine nowadays does not cover the period of time involved in the literal meaning of the word, and is usually, although not solely, carried out in respect of seagoing traffic. Ships which come from an infected port are required to lie out at sea, or in the port roadway, within a prescribed anchorage, until crew and passengers have been examined by the Port Sanitary Authority, and until—if sickness has occurred during the voyage—the ship and cargo have been disinfected. If, on the other hand, a clean bill of health be presented, and the number of days which have elapsed between the date of sailing from the infected port and the date of arrival at the quarantine station be in excess of the incubation period of the disease, the introduction of which is feared, all that remains to be done is disinfection of ship and cargo. If the number of days be less, and if a sick bill be presented, then the crew and passengers are not allowed on shore till the period of incubation is passed. It has been found desirable in populous places to provide retaining houses, which are practically quarantine stations, wherein to place persons occupying small houses in which infective disease has broken out, and where they may be retained, at municipal expense, until the disinfection of the infected premises has been overtaken, and until the incubation period of that disease is over. This, in our view, is an important item of sanitary equipment, for it deals with that population amongst which insanitary conditions most likely prevail, and which, therefore, demand most supervision.

Isolation is either effected at home or in special hospitals for the reception of the infected sick. The possibility of adequate isolation in the home depends (1) upon adequacy of room space; (2) efficient separation of sick room from the rest of the occupied rooms; (3) proper nursing by one whose sole duty is in the sick room; and (4) suitable means of disinfection of clothing during the currency of the illness. Under ordinary circumstances, it is impossible to have adequate isolation in houses of three apartments, or less. It is, then, a safer practice to send the patient to a isolation hospital. Even in larger houses the problem i difficult.

Disinfection, as the word implies, means the operations which are carried out against the infection (so as to destroy it), whether it be of the body of the infected person, his clothing, bed-clothing, or of the room he has occupied, and its contents. Efficient disinfection includes them all. From the imperfect carrying out of such measures in regard to any one of these, disease may be disseminated, and disinfective measures more usually err on the side of inefficiency than of over-carefulness.

### What Doctors Expect from Nurses.

On the 24th inst., at Toynbee Hall, Dr. Stephen Mackenzie delivered a lecture on the above subject to the members of the Toynbee Nursing Guild. There was a large attendance of members and friends of the guild, among the latter being Canon and Mrs. Barnett, Lady Vincent Barrington, Mrs. Stephen Mackenzie, Miss Rosalind Paget, Sir John Gorst, M.P., and Miss Wills and Mr. Winny, the hon. secretaries. Sir Vincent Barrington presided. Dr. Mackenzie pointed out that under varied conditions of nursing there were varied requirements, but the points he would bring specially under their notice that evening were the principles which should be mastered and kept constantly in view by every good nurse, whatever the conditions under which she might be working. These principles, he said, were that a nurse should be clean both in her habits and in her person; that she should be exactly truthful, rather confessing her ignorance on any subject than pretending to know, because she thought she ought to know; she should be quiet both in her manners and conversation, for nothing was more wearying to a helpless patient than to be tended by a bustling, harum scarum nurse, who thought that by hurrying over her work she could save a minute or two of time. She should also keep her virtues in the background, and not parade them for the patient's edification. A nurse should take care of herself, because on the maintenance of her health and strength depended her ability to do her best for the patient under her care. Of course, in institutions this was done for her, but in private houses the nurse must look after herself, and although it might, when exalted to a pinnacle of authority, be an unpleasant thing to draw attention to the fact that one's powers are limited, it must be done, for a nurse was often tempted to overtax her powers of endurance, and to give up her necessary rest for what might seem the good of her patient, but which in reality would be the reverse. One of the things to be rigidly attended to by a nurse in this respect was, Dr. Mackenzie pointed out, the presence of any open wound on her hands, when every care should be taken to keep such sore places covered. As regards the nurse's relations to the doctor she should be strictly obedient to his instructions, minutely exact, and keenly watchful. The doctor depended to a great extent on the nurse's observations, and where these were made with intelligence much might be done by her to assist him to do the best for the case. Dr. Mackenzie acknowledged his indebtedness to many nurses, whose trained watchfulness had been of the utmost value to him in dealing with cases; although the nurse's sphere of activity was not the same as the doctor's, yet it was quite of equal importance, and of her much was required in the noble calling which any woman might be proud to follow, and the value of which was more recognised at the present time than it had ever been in the past.

In a humorous little speech Sir John Gorst, M.P., proposed a vote of thanks to Dr. Mackenzie on behalf of the guild, and Sir Vincent Barrington followed with a few concluding remarks.

The next lecture will be on December 29th, when Miss Helen Webb will address the members on "The Management of Infants."

### Minor Appointments.

Hackney Union Infirmary.—Miss Eleanor A. Harvey has been appointed Assistant Matron at this infirmary. She received her training at St. Olave's Infirmary, Rotherhithe.

Royal Berks Hospital, Reading.—Miss Mary Smith has been appointed Charge Nurse at this hospital. She was trained at King's College Hospital and at the Evelina Hospital for Children, and recently for two years held the position of out-patient sister at the Children's Hospital, Chelsea, where she also took three months' holiday duty as home sister.

Fulham Union Infirmary.—Miss Isabella Nicoll has been appointed Superintendent of night nurses at this infirmary. She has worked as charge nurse at the Eastern and the North-Eastern Metropolitan Asylums Board fever hospitals, and held a similar position at the Scarborough Hospital and Dispensary. Miss Nicoll has also had a year's experience in private nursing in connexion with the General Nursing Institute, Mandeville Place.

Croydon Union Infirmary.—Miss Ridal was unanimously appointed to the position of Head Nurse at this infirmary on Tuesday, November 17th. Miss Ridal began nursing as a pupil probationer at the East-end Lying-in Home, London, afterwards going for three years' general training to the Sheffield Union Infirmary. She was then appointed charge nurse at the Gore Farm Small-pox Hospital, being transferred to Winchmore Hill when the former hospital was closed. Miss Ridal has since had seven months' experience in district work under the Paddington and Marylebone District Nursing Association, and she holds the diploma of the L.O.S.

### Nurses in 1896—Their Quarters, Hours, and Food.

[These articles exhibit the actual condition of affairs in the spring of the present year.]

### QUEEN CHARLOTTE'S LYING-IN HOSPITAL.

### I.—Terms of Training for Midwives and Nurses.

The conditions of training in midwifery and monthly nursing which prevail at Queen Charlotte's Hospital may be taken as fairly typical of lying-in hospitals as a class. A progressive step has lately been made in raising the shortest period of training for monthly nurses at this institution to three months; formerly two were allowed to suffice. We are frequently asked by correspondents where "free training in midwifery and monthly nursing may be obtained," and may take this opportunity of stating that no lying-in hospital gives free training. The fees, including board and lodging, for three months' training in midwifery at the London lying-in hospitals varies between twenty-one guineas at the City of London Lying-in Hospital to twenty-five guineas at Queen Charlotte's and the General Lying-in Hospital, Lambeth. Fifteen guineas is the sum paid for three months' training in monthly nursing at Queen Charlotte's Hospital.

Candidates for midwifery training at Queen Charlotte's must be not under 23 nor over 40 years of age. A registration fee of one guinea has to be sent with the application, which is deducted from the fee for the course when the pupil enters the hospital, or is returned to her if the application is refused. A personal interview with the matron is necessary before an application is accepted by the committee. Pupil midwives are given three weeks' training in monthly nursing before the course of instruction in midwifery is begun; instruction is given them by the sister midwives, who are trained nurses, in practical midwifery in the labour wards, and when doing duty in the out-patient department. They also receive instruction from the superintendent of the out-patient department and from the midwives of the districts to which they are appointed, and constant lectures are given by members of the medical staff. Each pupil is examined on the completion of the term of training, and "provided she proves herself competent to discharge the duties of a midwife will receive a certificate, but such certificate will not entitle her to undertake the medical treatment of cases, nor the management of complications in labour. It must be distinctly understood that they will not receive this certificate if found unfitted for the duties of a midwife at the end of their training." Pupils who fail to pass the examination for the certificate in midwifery granted by the hospital the first time may be allowed to present themselves for re-examination later on at the discretion of the examining physician. Fees, £26 5s. for three months, are paid to the secretary in advance.

Pupil nurses are subject to the same conditions as regards age limit and registration fee, and personal interview with the matron. Instruction in monthly nursing is given them by the matron and the sisters, and a certificate is given at the end of the three months' training to those who prove themselves competent. A nurse who has gained the certificate can have her name placed on the hospital Register of Monthly Nurses without charge.

The permanent sisters of the wards are required to have completed three years' general training and to hold the L.O.S. certificate.

### II.—Hours of Work and Times Off Duty.

The conditions under which pupil midwives and nurses work at a lying-in hospital are, of course, very different from those which obtain at an ordinary hospital, the hours on and off duty for the midwives especially varying with their cases. Day sisters' and nurses' hours of work are—approximately—for the former from nine a.m. to nine p.m.; and the latter, from seven a.m. to nine p.m. Night nurses are on duty from nine p.m. to nine a.m.; the night sister from nine p.m. to ten a.m. Two hours off duty, at the most convenient time, are allowed to all the staff every day, and the permanent staff—the sisters—have leave from six p.m. to half-past ten p.m. every other evening, and every other Sunday off duty, with an annual holiday of three weeks. Pupils do not have days or half-days during their brief stay in the hospital, during which period there is a great deal to learn, the midwives especially having, at its conclusion, to pass both the examination of the hospital and that of the London Obstetrical Society.

### III.—Meals.

Breakfast for the day staff is at half-past seven a.m., the nurses having previously had a cup of tea before going to the wards for half an hour. Dinner for the day staff is from half-past twelve to half-past one, tea from four to half-past four, supper eight to half-past eight p.m. Night sisters and nurses have supper at half past eight p.m., and dinner at eight a.m. The arrangements of old days when pupils at Queen Charlotte's slept in the wards with their patients, and prepared their breakfasts in the same place, have disappeared under the rule of Miss McCord, the present matron. All meals are laid in the dining-rooms, except the night nurses' ward meal, and tea, which are taken in what is called the "nursery," a small day-room or ward kitchen near the wards. The dietary has undergone considerable improvements. For breakfast, tea and bread and butter, eggs, bacon, or fish are provided; dinner consists of hot joints with vegetables and pudding; jam is supplied for tea on Sundays; and for supper soup or meat or pudding. Ale and milk are provided for dinner and supper.

### IV.—Salaries and Uniform.

The permanent staff of sisters or head nurses receive salaries varying from £25 to £40 per annum, with board, lodging, and washing. No uniform is given by the hospital.

Pupil midwives and nurses are required to provide themselves with the uniform of the hospital, consisting of four white piqué dresses, twelve large aprons of white linen, and caps of uniform pattern. None but these washing dresses are allowed inside the wards. Pupils are also required to pay for their own washing.

### V.—Quarters.

The accommodation originally provided for the midwives and nurses on the top floor of the hospital, must for a long time past have been entirely insufficient, and crowded and uncomfortable, at least for the pupils and nurses who slept two or three in a room, with very inadequate toilet arrangements. Now Increased accommodation is provided in a temporary Home—Sisters have bed-sitting-rooms, also on the top floor. There are two dining-rooms in the basement, dismal apartments, as all basement rooms are; but better time sare now at hand. The ground occupied by some houses formerly rented in the Marylebone Road for the use of the pupils has been acquired by the new railway, temporary quarters have been taken in Blandford Square, and a new nurses' home is about to be begun. The many friends and supporters of Queen Charlotte's Hospital ought to respond with alacrity to the appeal for funds to carry out well and thoroughly this needful work, for it is as incumbent upon a charitable institution to provide proper quarters for its own paid staff, and for the pupils who pay well for the training they receive under its auspices, as it is to care for the efficient treatment of the patients.

### A Book and its Story.

### A TANGLED GARDEN.

"A Tangled Garden"* has the agreeable characteristics of Mrs. Fred Reynolds' earlier novel, "Llanartis," and the authoress shows by her present literary contribution that she is by no means the writer of one book, but gives promise of even better work in the future. The scene of the story is laid in Wales, a country of which the authoress appears to have a personal knowledge, and the framework of her story (as, indeed, the characters set in it) are undoubtedly sketched from life, and, so far as the human element goes, from a somewhat saddened view of life. "A Tangled Garden" is the history of a man's folly, or rather of momentous circumstances therefrom ensuing, for Mrs. Reynolds concerns herself in these pages rather with Dennis Ackroyd's atonement than with past conduct of which it was the outcome. The prologue is the key to the story, as it is told in these pages:—

"Lorna, will you marry me?"

The speaker was a good looking young man with an attractive face. There were signs of power in the wide level brow and high forehead. . . . A keen observer would have considered Dennis Ackroyd's face pre-eminently fitted to express the passion and tenderness of love, and yet seen at a moment when one would have expected the most commonplace countenance to be fired and glorified, the well-drawn features looked strangely cold and impassive.

"Will you marry me?"

Before him, reclining amongst great silken cushions, dressed in the richest and daintest of tea-gowns, was a superb example of womankind. "Lorna Fortescue was already past her first youth, but had blossomed into a fuller, richer beauty than even her girlhood had promised. Lovelier than usual she looked just now. All the passion that was lacking in the young man's face was overflowing in her's; her eyes dark, glowing, rested with hunger on his face; a hunger that comes to the heart of every woman sooner or later, sometimes, alas! too soon, sometimes, God help her! too late; and whilst he is waiting for her answer the young man reviews the incidents of his past career as shared by Lorna Fortescue." He had met her in the whirl of London society. Among the fair women who moved in gay circles she, the woman he had just asked in a strangely constrained manner to marry him, had been the fairest. She was a widow, he was told. The charm of young Ackroyd's manner, his boyish innocence, pleased the faded taste of the society beauty. The interest they felt in each other strengthened into a closer intimacy, and now there was but one course open to a man of honour. He had fallen, but he was not degraded. Love was dead, but honour remained. And such is the purport of Dennis Ackroyd's thoughts as he stood sad, passionless, awaiting Mrs. Fortescue's answer to his question. It came at last, slowly; he could have almost thought unwillingly—"No."

"You will not?" "I cannot, Dennis. Because—because—" she rose and stood before him, and her face—the beautiful, passionate face, now trembling with emotion, was close to his—"because, Dennis, my husband still lives. . . . It was some time after we met before I realised that you believed my husband to be dead. And by that time I had no wish that you knew the truth." Then followed the woman's defence—a *mariage de convenance*, a neglectful husband, a heart which craved for affection, a union unblessed by children—"If," she explained, with reference to this, "I had had little children, I think I might have been a better woman, but even this consolation was denied me. I have never had anyone to love—till now."

And then, with forgiveness asked on the man's side and accepted on the woman's, the two part, whose fates had been so disastrously bound together.

Some years later we are introduced to Dennis Ackroyd in circumstances other than those with which he was earlier associated. It is in the heart of a mountain district in North Wales, when we come upon him again. He is leading a small boy by the hand, over whose welfare he evinces more than an ordinary interest. This visit on the man's part is due to a few lines which had chanced to catch his eye in a morning paper, wherein a widow lady offered a happy home to an orphan boy or girl. Dennis Ackroyd proceeded further into the matter, and after certain preliminaries had been gone through brings his boy to a new home.

Mona Romney, at whose house itis the home is offered, is a young widow, mourning the loss of her husband and child. Into the loneliness of the woman's life had come a great longing after the presence of someone—some child companion to care for and to live for. The idea of advertising a home ultimated in the appearance of Dennis Ackroyd and his boy Robin upon the scene. It is barely necessary for the reader to refer to the prologue for the key to the mystery surrounding this little fellow's existence. But it was some time before the man explains in any explicit manner the tragedy surrounding his boy's birth; and, meanwhile, the child had strangely endeared himself to the lonely woman. His advent had been the herald to her of a return to mother life, and it did not take long for Mona Romney to give her whole heart to her small charge.

Robin's father came and went on flying visits to the child's new home, and his interest in the woman increased with each fresh meeting; Robin was a common interest to both. Much of the best feature of the book centres round his little person, and he is described in a graphic and life-like manner. At length the *dénouement* of his parentage is forced upon Dennis, who confides his sin to Mona Romney. By this time it is no secret that the man is ardently attached to his child's guardian, but the woman's heart is unassailable and buried in her husband's grave. Little by little, after the shock of Dennis' disclosure is wearing off, she feels attracted by the man, feels an admiration and respect for his repentance and manly, honourable attitude towards the innocent object of the remorse. Robin's mother, it must be explained, was dead, had died at his birth, and Dennis' intentions are to treat the boy in every sense as his son. But fate stepped in, and one summer day little Robin is removed from Mona's care, having succumbed to a mortal illness, only too fortunately for himself before his short life was clouded by any unhappy revelations as to the history of his parentage being revealed to him. "And the child-spirit so unwillingly welcomed to its earthly dwelling, so little wanted, so early taken, so passionately loved and bitterly mourned, had not lived in vain."

His work was done.

So the book closes, having left an impression of melancholy upon the reader. The intention of the writer was, we venture to conjecture, directed otherwise, and her volume written to show in an allegorical sense that the human soul, like a "Tangled Garden," has capacities despite accumulation of its many weeds. Such doctrines are salutary, stimulating. The pity of it is that Mrs. Reynolds has not told her story in a brighter vein; a touch or two of humour, of vivacity on the writer's part, instead of lessening the pathos of her scheme, would have heightened it by a greater artistic contrast.

* "A Tangled Garden." By Mrs. Fred Reynolds. (London: Hutchinson and Co. 1896.)

### Midwifery Papers.

### XI.—PUERPERAL FEVER.

ONE of the most distressing and at the same time detrimental accidents in the practice of a midwife is an outbreak of puerperal fever. If puerperal fever develops in a case she is attending, the midwife should at once cease visiting all other cases, and on no consideration should she undertake a fresh confinement until she has undergone some weeks' disinfection and quarantine. Puerperal septicæmia is highly contagious, and is readily carried about from one lying-in woman to another. The tenacity of the poison, too, is so great that the ordinary means of disinfection by antiseptic baths and a complete change of clothing have been over and over again proved ineffective, and there seems no other course open to the midwife than complete isolation from all confinement cases for some weeks. Of course to most midwives this entails a serious pecuniary loss, but when it is remembered that undertaking a case while still infected may and will most likely mean the infection and death of the patient, no midwife who understands her duty dares run so great a risk.

Under these circumstances it is exceedingly surprising and shocking that so many midwives, utterly regardless, it seems, of the lives of their patients and their own reputations, recklessly undertake other confinements when a clear case of septicæmia has occurred in their practice. I can only believe that many of them do not realise the extent of the dangers they run and the harm they may do.

Puerperal fever is a dirt disease, and is generally caused by septic matter being conveyed to the patient during delivery on dirty hands or dirty instruments used by the attendant doctor or midwife. Owing to the open condition of great blood vessels over the placental site, and occasionally from laceration of the soft parts taking place during the birth and so exposing blood vessels, a newly-delivered woman is most highly susceptible to septic poison, and hence the necessity that not only should the surroundings of bedding and room be absolutely aseptic, but what is almost more important the clothing and hands of the nurse should be so too, for she is the most dangerous source of infection, owing to the nature of her duties.

SYMPTOMS.—The symptoms of puerperal fever generally show themselves on the third day after delivery. The first signs may be restlessness and a quickened pulse, followed by a rigor with a sudden rise in temperature and a severe headache. Suppression of, or change in the character of, the milk and lochia also takes place; the lochia generally becomes highly offensive in odour. The tongue becomes fur-coated and the breath has a curiously sweet smell. As the disease develops the rigors increase in intensity, and the pain and tenderness on pressure at first felt over the uterus extends over the abdomen, which becomes much distended through the collection of flatus in the intestines. The patient lies on her back, with her knees drawn up, and her face is sunken and assumes an anxious expression. The distended abdomen is generally so exceedingly painful that a bed cradle has to be used to remove the pressure of the bedclothes. Very often there is diarrhœa and vomiting of dark matter like coffee-grounds in appearance. The temperature is jerky, sometimes reaching 106 deg., at others falling below normal. Puerperal septicæmia is occasionally accompanied by great pain and swelling in the joints. When the patient becomes much exhausted delirium sets in, and there may be retention of urine, necessitating passing the catheter. Puerperal fever cases vary in intensity; some run a rapid course in a week, ending in death, while others may linger on for some time, the patient dying at length from exhaustion. The suffering is always great, and in the rare cases of recovery the patient will most likely remain delicate for life. A midwife cannot be too careful that no precaution is neglected by which occurrence of such cases can be prevented in her practice.

TREATMENT.—Send for a medical man. Do not wait to be sure, but send at once if the temperature rises or the abdomen is tender. The doctor will most likely give an intra-uterine douche, so have hot water in readiness. Unless specially ordered, a midwife should never take upon herself to give an intra-uterine; the dangers are great from collapse of the patient through shock from a carelessly given douche. The patient should have strong beef-tea, egg lightly beaten in milk, milk and soda, and a stimulant, if ordered by the doctor, given at short regular intervals, and everything should be done to keep up her strength. After the profuse perspirations and rigors, she should be gently sponged under a blanket with hot water, and a warm, dry nightdress should replace the damp one. In doing this, and in changing her sheets, the patient must be moved as little and as gently as possible. The back and hips will need special attention, for bed sores form on the slightest pressure in a septic case with a high temperature. In giving a vaginal douche, or in passing the catheter, be very gentle, for the parts will most likely be very sensitive and painful. Before passing the catheter, do not forget to sponge the external genitals with some antiseptic, so as to completely cleanse them from any discharge. For this cotton wool should be used, and then immediately burnt. Carefully cleanse the mouth and tongue at intervals with a solution of boracic acid or glycerine and borax. If poultices are ordered for the abdomen, they should be lightly made and changed often. They can be easily kept in place by a binder, which should not be too tightly applied. The nursing is all important, and nothing should be neglected that can help or relieve the patient.

RETAINED PLACENTA.—Sometimes blood-poisoning is set up, not from infection by some septic germ introduced into the uterus during the confinement, but by the retention of some portion of the placenta, which remains adherent to the uterine wall, and which gradually decomposes and sets up a general septic condition. Adherent pieces may be retained from careless attention during the birth, and especially if the placenta is not very healthy, and is friable or easily broken up. Pulling on the cord to hasten the birth of the placenta is one of the chief causes of this accident. Only careless midwives, and those who do not know their work, ever attempt such a dangerous proceeding as this, which may result in severe hæmorrhage from the sudden emptying and non-contraction of the uterus, or may cause inverted uterus. From this accident the patient may die of shock, or hæmorrhage, and, of course, the risk of blood-poisoning is greatly increased. But the most usual result of pulling on the cord is tearing away the placenta and leaving a portion behind. If the careless midwife neglects to examine the placenta, and so fails to notice that a portion is absent, then it is most likely retained till it sets up septic mischief by decomposing.

TREATMENT.—The same as in an ordinary case of puerperal septicæmia. A doctor must be summoned, and what is left of the placenta removed.

PHLEGMASIA-DOLENS, or white leg, is another complication of the puerperal state. It is caused by the formation of thrombii in the veins of the legs. The first symptom is acute pain in the calf, followed by swelling of the affected limb, which may become hard and tense and of a shiny white colour. The pulse and temperature rise, the tongue becomes glazed or coated with fur, and the bowels are generally constipated.

TREATMENT.—The patient must be kept in bed and the bowels be relieved as soon as possible. Poultices or hot fomentations, sprinkled with laudanum, should be applied to the limb. A small water-pillow, very slackly filled with hot water and placed under the limb, has been found very useful in giving support and heat to the affected part. The water should be renewed from time to time. A cradle should be placed over the leg to support the bed-clothes, for the slightest pressure increases the pain. In a case of phlegmasia-dolens a medical man should be summoned.

Puerperal Eclampsia.—This dreadful disease is one of the most formidable complications in widwifery. It is a kind of epileptiform convulsion, which is accompanied by albuminuria, and is generally supposed to be caused by it. Eclampsia may occur in pregnancy, generally in the latter months, or during or after labour. It is more often associated with first pregnancies, and sets in about twelve hours after labour.

Symptoms.—An attack sometimes occurs without any premonitory symptoms, but more frequently it is heralded by severe headache, dimness of sight, nausea and vomiting, pain in the chest, œdema of face and limbs, irritability, and a general feeling of malaise. Œdema of the face or upper extremities should lead one to suspect kidney mischief, and the urine should be examined by a doctor, and the case, if albumen is present in large amount, at once given up to him. But if a midwife arrives when a patient is having a fit, she must remain and do the best she can while awaiting a doctor. During the attack the face becomes livid, tonic and clonic spasms of the muscles occur, the muscles of the face twitch violently, the eyes turn upward, so that only the whites are visible, the tongue protrudes and very often gets seriously bitten during the fit if care be not taken to keep the teeth apart, and the mouth is drawn on one side. Respiration is arrested temporarily at the outset of the attack, and is followed by hurried and irregular breathing marked by a peculiar hissing sound. The attack lasts only a few minutes, and is followed by partial coma, with stertorous breathing. The attacks may follow each other very quickly, or an interval of several hours may elapse before a second occurs.

Treatment.—If eclampsia sets in during pregnancy premature labour is induced by the nervous shock. The uterus should be emptied as soon as possible, so directly the placental membranes can be reached they should be ruptured. Give an active purgative, and see that the bladder is emptied; if possible avoid the use of an enema or catheter, as interference in this way may induce a fit. Send for the medical man at once, and keep the patient as quiet as possible in a darkened room. The mortality in eclampsia is high, and recovery or death generally depends on the severity of the paroxysms. The dangers to both mother and child are, of course, great. The duration of eclampsia is generally about thirty-six hours, and is ended by the death or recovery of the patient.

### Gloucester District Nursing Society.

We have received the report of the Special Committee appointed by this Society early in the present year to organise the work of nursing small-pox patients in their own homes, a work which the General Committee of the Society decided to offer to undertake, "whatever might be the pecuniary risk," when it became evident that the hospital accommodation at the disposal of the Sanitary Authority was altogether insufficient for the emergency. Its pages are full of interest, showing as they do how splendidly the Society answered to the terrible strain upon its resources, and how admirably organised and carried out were all the arrangements made to cope with the epidemic. The Special Committee, composed at first of Dr. R. W. Batten, the Rev. A. C. Eyre, Mr. H. E. Waddy, and Mr. G. Whitcombe, and afterwards further reinforced, worked with a will. Miss Evans, the superintendent, herself undertook the management of the small-pox nursing; five houses were secured for the special staff, consisting of 24 trained nurses, augmented by some 60 assistant nurses, men and women, and the task of combating the progress of the disease was begun in earnest. During the four months of the epidemic the nurses attended to 946 persons. The committee undertook to provide any relief required during the first twenty-four hours of attendance upon a patient, after which time the Guardians took up the case, a system of mutual returns being arranged so that overlapping and wasting of relief might be obviated. The Committee of the Sanitary Authority and the Guardians rendered every greatest gratitude for assistance in a time of dire need of which it would be impossible to overestimate the value. The report states that "The thanks of the whole community are assistance to the Society, to which, indeed, they owe the due to Miss Evans . . . for the zeal, energy, skill, and devotion with which she threw herself into the work," and points out the pleasant fact that with a fluctuating staff, varying from 50 to 60 persons, not the slightest rub or friction occurred during those months of unremitting labour. The committee may well hold that the society must feel under the greatest obligation to Miss Evans for proving that it was capable on a sudden emergency of thus successfully undertaking a great work, and so justifying the confidence placed in it by the public. Warm thanks are also due to the honorary matron, Miss Julia Helps, who "at a moment's notice dissevered herself from her family and friends in order to help Miss Evans to stem the terrible first rush of cases," and continued her services for four months. The Society received altogether from the public in donations £3,556 13s. 3d., making, with £20 5s. 4d. interest from bankers, a total of £3,576 18s. 7d. The Special Committee were fully prepared, had it been necessary, to extend their organisation into country districts, and made every effort by the issue of circulars suggesting the proper steps to be taken in the event of an outbreak of the disease in any parish, to encourage a state of preparedness which might nip it in the bud. The Gloucester District Nursing Society has merited the grateful thanks of the whole city.

### Trained Nurses' Club.

In spite of a very wet afternoon, a number of visitors found their way to the annual sale on behalf of the Trained Nurses' Club (12, Buckingham Street), held on Wednesday in this week at Mrs. Lorent Grant's house in Nevern Square. There were many pretty and attractive things on the various stalls, and the briskness of the business going on seemed to promise that not many would be left on hand by the end of the day. The club stall, furnished entirely by members' contributions, was presided over by Mrs. Nichol, the secretary, and the "Nursing Notes" stall by Miss Brierly. An entertaining programme of music and recitations was carried through by the help of several kind friends.

### Appointments.

International Hospital, Naples.—Miss Moore, whose appointment as Matron at this hospital we announced some time back, asks us to correct a slight mistake in the notice, wherein her Christian name was given as Mary instead of May. Miss Moore also informs us that she was probationer, not staff-nurse, at the West London Hospital, and that after being staff-nurse at Sheffield Infirmary she subsequently held the post of sister there for three years.

### The Caged Orphans at Broadstairs.

Mr. T. F. Raven writes: The charges which have been preferred by your anonymous "Medical Correspondent" and supported by yourself against St. Mary's Orphanage, Broadstairs, amount to this: (1) that the children are shut up during the night in cages like those used for wild beasts in travelling menageries; (2) that from the culpable carelessness

Cubicles at St. Mary's.

of the Sisters of the Church the children are in danger of losing their lives in the event of fire owing to these sleeping arrangements. In support of the first of these charges a "rough" sketch of some cubicles in use at Kilburn was published in The Hospital of October 24th; and in support of the second your correspondent stated that before the children could be released from the cubicles each of them must be opened singly. As to the wild beast cages, I have branded that slander as it deserved to be branded, as wilful and dishonest. The statement that the cubicles opened singly was a stupid mistake, and your correspondent has been obliged to confess his blunder. As, however, the charges in the main have not been withdrawn or apologised for, I have taken further steps to vindicate the Sisters of the Church and myself. I, therefore, forward you a photo block of two of the cubicles at St. Mary's of average dimensions and appearance, which I request you to print in the next issue of The Hospital. Your readers will then have the opportunity of seeing for themselves how much justification there was for your anonymous attack, and how far your rough sketch is a faithful representation of "cages similar to those at St. Mary's." I have also to state that on the night of November 4th I attended a fire drill at the orphanage. The children were asleep in their cubicles, and the Sisters were placed in their ordinary situations for the night. Two wards, containing ninety children, about equally divided in point of numbers, were aroused. In order that we should be at no unfair advantage, one of these wards contained the youngest children. The ward where the elder children slept was one storey above it. The fireman, Mr. Pemble, gave the alarm, and I remained downstairs to time the experiment. From the time that the whistle sounded to the time when all the children (ninety) were down on the ground floor was just under four minutes. The first baby was down in fifty seconds, carried by one of the Sisters. There was no panic, only one child cried a little, and all were quite well next day. Mr. Pemble had made the same experiment previously with the same result, and he, as a practical fireman, is of opinion that, in the event of fire, the cubicles are far superior to open beds, and enable those in charge of the children to control and guide them to safety. As I have neither time nor inclination for further correspondence on this subject I will make my final rejoinder to your "Medical Correspondent." He seems to have just discovered that his honour has been impugned. Most certainly it has, from the first. "Wilful misrepresentation" carries dishonour with it, and that is what I have fastened upon him. He declares that the language which he employed does not convey the imputations which have been deduced from it. But when a man states that certain cubicles are like wild beasts' cages he implies, if language means anything, that the children who sleep in them are treated, so far as their sleeping arrangements are concerned, like wild beasts. If he did not mean what he said, well and good, but let him say so and apologise for what he has said. As to the "sound and fury" of my letters, and as to my "lashing myself into a fine fury" and all that nonsense, I shall not waste my time in answering it. I am quite satisfied to leave the whole correspondence to the judgment of any impartial reader. Of yourself I will ask one question. If such a system exists (perhaps it does not exist) as the ethics of journalism, I should like to know whether it is laid down that anonymous attacks upon individuals or institutions are permissible in respectable journals? To the non-journalistic mind the answer seems fairly obvious, but one would like a ruling on the point *ex cathedrâ*. I have no more to say in the matter of this correspondence beyond congratulating your correspondent upon his well-sustained

Copy of a Portion of the "Sketch of Similar Cubicles at Kilburn," which appeared in our Issue of October 24th, omitting such parts as do not appear in the above Photograph.

anonymity. You always have the opportunity for the last word. Pray avail yourself of it.

⁂ The above is the photo-block furnished by Mr. Raven. It will be observed that it only shows the front of the two cubicles, the divisions, as well as most of the back, being hidden by what apparently are sheets or curtains, pictures, &c. From the photographer's point of view this certainly adds to the effectiveness of the picture, but it tends much to hide the cages themselves. To show how little use such pictures are, as refuting in any way what has been stated in The Hospital, we have had our "rough sketch" of the Kilburn cages cut down, so as to leave out as nearly as possible those details which are left out in the photo-block sent by Mr. Raven, and we think that a comparison of the two will show how very similar the Broadstairs "cubicles," so far as they are shown in the picture, are to the same parts of the Kilburn cages, as shown in our "rough sketch." As regards Mr. Raven's letter, and his peculiarly innocent question whether "anonymous attacks on individuals or institutions are permissible in respectable journals," one is inclined to wonder whether he ever reads a newspaper and what he thinks are the aims and objects of journalism. All public institutions, and all those who occupy official positions in them, are open to public criticism, and anyone who reads the leading papers of the day must know that many of the most effective attacks on abuses in public institutions have been made by anonymous journalists, and that the most drastic reforms have resulted from such attacks. In this case no individual was attacked. We received a letter from a correspondent, in whose word we had confidence as to the existence of a great abuse in a public institution, supported by public subscription. We published that letter, and then Mr. Raven intervened. But this was done of his own free will, and he has no excuse whatever for saying that there was any anonymous attack upon an individual. There was a public indictment of what we considered to be a gross abuse in a public institution, and probably most people will be of opinion that Mr. Raven would have done better if he had tried to remedy the abuse instead of striving to find out the name of the informant. We need hardly say that no serious attempt has been made to traverse the statements made in the origina letter of our "Medical Correspondent." One error has certainly been pointed out. Whereas our correspondent said that "no escape would be possible for the unhappy inmates of the cubicles until the doors had been one by one unfastened," Mr. Raven says, "This is not the fact; a large number of them open at once by a very simple mechanical contrivance. Those that do not open in this way can be rapidly unfastened by any of the sisters." He fails, however, to say how many are opened at once and how many have to be opened singly, and it is fair to presume from the tone of his letters that had this been a point really worth anything he would have been glad enough to have told us the number and to have convicted our correspondent with something more than vague generalities. "Nurse A. B.," however, writing on the same subject, and also in defence of the sisters, admits that the cubicles in the infirmary and some in the babies' dormitory do open one by one "as he describes," while the rest are opened apparently not one by one but row by row, and states that in case of fire the "sisters could release the children at their discretion." The fact, then, that the children are locked up in wire cubicles, which we do not hesitate to describe as "cages," is admitted; the fact that a large number of these cages (and those where the most helpless children are lying) have to be opened one by one is also admitted; and the fact that in regard to the rest they can only, even in case of fire, be released at the "discretion" of the sisters is also admitted. This ought to be sufficient for the public, and we may be sure that a fair-minded public will not allow its indignation at these proceedings to be lessoned or its attention to the point at issue to be diverted by Mr. Raven's ingenious attempts to "get at" the correspondent who exposed these abuses.

Mr. Hugh P. G. Maule, architect, Great Marlborough Street, writes: As the correspondent who sent you the original sketch of the cubicles at Kilburn, which appeared in The Hospital for December 1st, 1894, I have been much interested in the recent correspondence in your columns under this heading. In November, 1894, I visited the orphanage at Kilburn, and from careful observations made the sketch which you published, and which is an accurate representation of the cubicles as they then existed. I do not know if they have since been altered. In many instances the cages were placed together in rows, and the doors were locked by a catch on the long iron rod, worked by a lever at the end as shown in the drawing. The doors being once closed, and the lever shut down, the children could not have got out of the cages unless the rod was again turned by outside agency. I think any unprejudiced person, seeing the actual cubicles would admit that, if anything, the drawing does not make them out as evil looking as they are.

### Everybody's Opinion.

### MELBOURNE HOSPITAL.

Miss M. D. Farquharson, Lady Superintendent of the Melbourne Hospital, writes: Kindly oblige me by correcting a statement made in The Hospital for September 19th, namely, that "many of the best nurses now at the Melbourne Hospital trained under Miss Farquharson," the fact being that all my present nurses trained under my predecessor, Miss Rathie (Mrs. Cutts). I have been in my present position barely a year, and none of my nurses at the Alfred Hospital left there to follow me, nor servants. I should certainly have discouraged it had such been the case.

⁂ We have much pleasure in publishing the above letter, and regret that any error should have crept into our description of the Melbourne Hospital, which was based upon information supplied to us from an authoritative source. —Ed. *T. H.*

### Where to Go.

Royal British Nurses' Association.—The Secretary requests us to state that the course of demonstrations on Invalid Cookery, now in progress at 17, Old Cavendish Street, W., will be continued as follows: Tuesday, December 8th, half-past two p.m. (sixth lesson)—Roast chicken, bread sauce, stewed oysters, bread and butter pudding, lemon sponge, cornflower blancmange, rice jelly. Tuesday, December 15th, half-past two p.m. (seventh lesson)—Restorative soup, boiled sole, melted butter, sponge cake, lemon jelly, linseed tea, arrowroot. The course will be resumed on Tuesday, January 12th, 1897, after the Christmas recess.

### Notes and Queries.

The contents of the Editor's Letter-box have now reached such unwieldy proportions that it has become necessary to establish a hard and fast rule regarding Answers to Correspondents. In future, all questions requiring replies will continue to be answered in this column without any fee. If an answer is required by letter, a fee of half-a-crown must be enclosed with the note containing the enquiry. We are always pleased to help our numerous correspondents to the fullest extent, and we can trust them to sympathise in the overwhelming amount of writing which makes the new rules a necessity. Every communication must be accompanied by the writer's name and address, otherwise it will receive no attention.

*Holt-Ockley System.*

(53) Where can I obtain information about cottage nurses and the Holt-Ockley system?—*E. A. H., Carlisle.*

Write to the hon. secretary of the Ockley Nursing Association, Mrs. Henry L. Steere, The Cottage, Ockley.

*Mistaken Impressions of Prison Life.*

(54) I see in the article on the above subject in The Hospital for November 7th reference is made to a paper by the Howard Association, "Charles Dickens' Prison Fictions," to a letter from the secretary of the association to the *Times*, and to another article in "a high-class periodical." Can you tell me how I can obtain these papers and articles? —*N. G. Mitchell-Innes.*

Write to the secretary of the Howard Association, Mr. W. Tallack, 5, Bishopsgate Street Without, E.C., for the information you require. See notice at head of this column regarding replies by post.

*Up-Country Nursing Association.*

(55) Addre s of this association wanted by Sister Knox, "A. R.," and "Enquirer."

We have given the address many times in this column. Hon. secretary, Major-General Bonus, R.E., The Cedars, Strawberry Hill, S.W.

*Orphans Caged.*

(56) Can you tell me the date of the supplement to *Truth* which dealt with the whole matter of the Kilburn Sisterhood, and where it could be obtained?—*Trained Nurse.*

A correspondent informs us that the supplement of June 18th of this year, can be had from John Kinsit, 18, Paternoster Row, price 3d., post free.

*Royal Scottish Nursing Association.*

(58) Can you give me any information respecting the Royal Scottish Nursing Association, Edinburgh?

Write to Miss Grant, 69, Queen Street, Edinburgh.

## Figures and Tables

**Figure f1:**
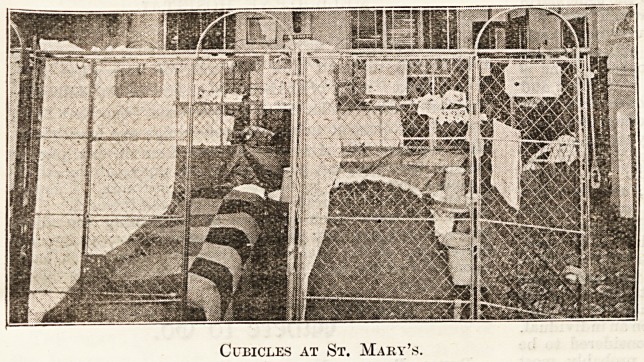


**Figure f2:**